# Assessing the Influence of Propofol Sedation Versus Propofol-Free Sedation on Recovery From Sepsis and Septic Shock in Mechanically Ventilated ICU Patients: A Retrospective, Propensity-Score Matched, Cohort Study

**DOI:** 10.7759/cureus.116479

**Published:** 2026-09-18

**Authors:** Lucas Luong, Sarah Vincze, Sarah Jacobson, Marioghae Otuada, Stephen Thompson, Aseel Walker, Dakota Balkir, Dhamodaran Palaniappan

**Affiliations:** 1 University of Connecticut School of Medicine, UConn Health, Farmington, USA; 2 Integrated Anesthesia Associates, Hartford Hospital, Hartford, USA; 3 Research Administration, Hartford Healthcare, Hartford, USA

**Keywords:** critical care, mechanical ventilation, propofol, sedation, sepsis, septic shock

## Abstract

Background: Propofol-mediated immunosuppression has been postulated in preclinical research, yet translational clinical evidence is lacking. We, therefore, retrospectively evaluated the influence, if any, of propofol versus non-propofol sedation on critical care requirements, prognosis, and risk of re-infection among mechanically ventilated patients with sepsis or septic shock.

Methods: This single-center, retrospective cohort analysis included adult patients admitted to any ICU at our institution from July 2017 through December 2022 with a diagnosis of sepsis or septic shock who required mechanical ventilation with sedation. Patients who received propofol sedation with or without* *other means of non-propofol sedation were compared to patients who received non-propofol sedation only. Univariate analysis was used to compare the primary outcome, ICU-free days, and secondary outcomes including intubation and mechanical ventilation-free days, infusion vasopressor support, and antimicrobial medications. Incidence of combined or hospital 30-day mortality and sepsis recurrence within six months were also compared. Time to infection clearance from blood was reported, as available. Total duration and infusion rate of propofol sedation were compared between patients who expired versus survived and between patients with versus without six-month sepsis recurrence. One-to-one propensity-score matching (PSM) was applied using sepsis diagnostic status, age, sex, BMI, and chronic comorbidity burden as covariates to manage confounding effects and identify comparable subpopulations.

Results: A total of 615 patients were included, comprising 511 (83.1%) propofol-sedated patients and 104 (16.9%) non-propofol-sedated patients. Prior to PSM, propofol sedation was more common among younger patients (median (IQR) years, 62 (50-72) vs. 66 (58-75), p=0.021). Patients treated for septic shock comprised most of the dataset (n=498, 81.0%). The non-propofol group received fentanyl sedation more commonly (84 (80.8%) vs. 234 (45.8%), p<0.001), though the median duration of fentanyl sedation was longer in the propofol group (1.9 (0.9-4.2) vs. 1.5 (0.4-2.8) days, p=0.012). Median ICU-free days were similar between non-propofol and propofol-sedated groups (median (IQR) days, 6.3 (2.1-14.7) vs. 6.8 (3.3-13.3), p=0.303). The proportion of patients requiring three or four pressor infusions was significantly greater in the non-propofol group (32 (30.8%) vs. 110 (21.5%), p=0.041). The combined hospital or 30-day mortality rate was also higher in the non-propofol group (45 (43.3%) vs. 165 (32.3%), p=0.031). After PSM, fentanyl sedation remained more common in the non-propofol group. Median ICU-free days remained similar between groups, but there were no longer significant differences in extent of vasopressor support required or mortality rates.

Conclusions: Propofol-containing sedation was not associated with fewer ICU-free days, poorer prognosis, or greater rate of re-infection among mechanically ventilated patients with sepsis and septic shock. Residual confounding and limited precision preclude exclusion of clinically important benefit or harm. Randomized controlled research is necessary to elucidate the clinical impacts of propofol sedation among mechanically ventilated patients with sepsis and septic shock.

## Introduction

Propofol is a potent anesthetic agent commonly used to induce and maintain anesthesia and for sedation in the ICU due to its favorable pharmacokinetic profile as well as its anxiolytic, antiemetic, anticonvulsant, and neuroprotective properties [1]. Among patients with sepsis or septic shock who require mechanical ventilation, propofol is the preferred choice for sedation due to its rapid onset and ease of attaining optimal sedation [2]. Nevertheless, propofol has been implicated as an immunosuppressive agent in several relevant preclinical studies, raising concerns for its use among patients with systemic infections in critical condition. One preclinical study evaluated the effects of propofol exposure in vivo on bacterial burden in *Listeria monocytogenes-*infected mice and found that propofol exposure increased bacterial burden by 10,000-fold compared to control-infected mice [3]. Consistent with this finding, additional preclinical studies have correlated propofol exposure with enhanced microbial growth of *Escherichia coli* (*E. Coli*) and *Candida albicans* (*C. albicans*) in vitro [4], increased dissemination of methicillin-resistant *Staphylococcus aureus* (MRSA) infection, and increased morbidity and mortality in in vivomodels of sepsis [5,6]. Impaired neutrophil phagocytosis and inhibition of key pro-inflammatory cytokines including tumor necrosis factor-alpha (TNF-α), interleukin-six (IL-6), and interleukin-one-beta (IL-1β) are commonly postulated underlying mechanisms of propofol-induced immunosuppression [3,5,7,8]. Suppression of effector T-cell-mediated immune response following propofol exposure has also been reported in vitro [9]. Propofol has also been indirectly tied to immunomodulatory effects via its lipid emulsification requirement for standard intravenous delivery; existing research suggests that lipids alter cell membrane fluidity, subsequently interfering with immune cell-bacterial interactions, among other postulated mechanisms [10]. 

Although a plethora of preclinical evidence suggests that propofol sedation may impair immune function and enhance microbial growth, it is unknown whether these findings translate clinically. More than 20% of patients with sepsis typically require invasive mechanical ventilation, which usually necessitates the use of appropriate sedatives [11]. Given the paucity of existing clinical research, it remains unclear whether mechanically ventilated patients sedated with propofol experience prolonged time to recovery and poorer prognostic outcomes compared to those who do not receive any propofol sedation. 

This study, therefore, aimed to retrospectively evaluate the influence of propofol sedation, if any, on critical care requirements, prognosis, and risk of infection recurrence among mechanically ventilated patients with sepsis and/or septic shock. The primary outcome comprised ICU-free days. Secondary, exploratory outcomes included intubation and mechanical ventilation-free days, extent and duration of infusion vasopressor medication requirements, antimicrobial-free days, frequency of combined hospital or 30-day mortality, time to infection clearance from blood, incidence of sepsis recurrence within six months, and time to sepsis recurrence. We hypothesized that patients who received propofol sedation had fewer ICU-free days compared to those who received alternative sedation without propofol exposure.

## Materials and methods

Study design

This single-center, retrospective cohort study included adult patients admitted to any ICU at Hartford Hospital (Hartford, CT, USA) for treatment of sepsis with or without septic shock between July 1, 2017, and December 31, 2022. This study (HHC-2023-0008) was approved by the Hartford HealthCare Institutional Review Board (IRB) (January 12, 2023); given minimal risk to participants, the requirement for written informed consent was waived. This study was performed in accordance with ethical standards set by the IRB and the most recent Helsinki Declaration of 1975. This study is compliant with the Strengthening the Reporting of Observational Studies in Epidemiology (STROBE) guidelines [12].

Study Population

Adult patients between 18 and 89 years admitted to the ICU with a diagnosis of sepsis with or without septic shock, requiring mechanical ventilation and infusion-sedation, were included. Patients in the ICU who presented with new-onset sepsis or septic shock and had not received any sedative infusions prior to diagnosis were also included. Diagnoses of sepsis and septic shock during the study period were identified using a predetermined list of sepsis-specific ICD-10-CM codes [13] (see Appendix A).

All eligible cases were then stratified into two groups based on the presence (propofol group) or absence (non-propofol group) of propofol sedation.

Propofol group: Patients who received any amount of infusion propofol sedation for any length of time, with or withoutother means of non-propofol infusion sedation following sepsis diagnosis. This group includes patients who were initially sedated with propofol, then transitioned to other means of sedation, and vice versa.

Non-propofol group: Patients who received any amount of non-propofol infusion sedation only, including dexmedetomidine, ketamine, and/or benzodiazepines as well as analgesia-first sedatives (fentanyl and hydromorphone), for any length of time following sepsis diagnosis.

Patients were excluded in the following circumstances: no infusion sedation during ICU stay, transfer from an outside ICU with a known diagnosis of sepsis or septic shock, expiration within 48 hours of mechanical ventilation start, comfort measures only sedation, or sepsis recurrence. Sepsis recurrence was defined as re-emergence of sepsis or septic shock within six months of a former encounter. Cases of recurrent sepsis were excluded to account for potential confounding influence of sepsis-induced immunosuppression and post (-sepsis) intensive care syndrome, which have each been associated with prolonged ICU and hospital durations and greater incidence of mortality in subsequent hospitalizations for sepsis [14-16].

Data collection

All data were gathered retrospectively from electronic medical records stored in our institution’s electronic privacy information center; no data were collected prospectively.

Demographics and Baseline Characteristics

Patient demographics (age, sex, ethnicity, race, BMI), comorbidity burden, and other relevant clinical information at the time of hospital admission were collected. The Charlson Comorbidity Index (CCI), a validated prognostic tool that assigns weighted point values to major medical comorbidities to estimate overall burden and severity of chronic disease, was used to quantify total comorbidity burden at the time of hospital admission [17]; it is specifically calculated by summing all points to attain an overall CCI score, where a higher score indicates more severe comorbidity burden. Frequencies of sepsis versus sepsis plus septic shock diagnosis were identified using the following ICD-10-CM codes: R65.21 and T81.12 (XA, XS, or XD). As available, frequencies of each microbe type identified from the first positive blood culture (gram-positive, gram-negative, fungal, or multidrug-resistant (MDR)) were reported per group. MDR infection status was defined as pathogens that display resistance to multiple antibiotics, including vancomycin-resistant *Enterococcus*, MRSA, and any *Pseudomonas* species. Cases in which blood cultures were taken, but no positive blood culture was identified, were reported as such. Rates of identifiable infection sources were reported, including the following categories: respiratory, urinary tract, gastrointestinal, skin and soft tissue, direct (device or line-associated), endocarditis, osteomyelitis, central nervous system (CNS). Infection source data were reported based on available positive culture data, consistency in identified microbe(s) between blood and source cultures, and available clinical documentation; cases in which blood cultures were taken, but no positive source culture was identified, were reported as such.

Antimicrobial medication use during hospitalization was compared between groups, broken down by pharmaceutical class (antifungals, beta-lactams, fluoroquinolones, macrolide antibiotics, and miscellaneous anti-infectives). Use of lipid emulsions, including total parenteral nutrition and/or Clevidipine infusions, disregarding propofol lipid emulsions, was also reported to account for potential confounding influence, given a surplus of evidence associating lipid emulsion with immunomodulatory effects [10]. Incidence of positive COVID-19 lab result taken during or within two weeks prior to hospital admission, as well as suspected or known clinical diagnosis of COVID-19, were reported to account for potential confounding influence on reported outcomes; reported frequencies were calculated using only sepsis encounters that occurred between 2020, the onset of the COVID-19 pandemic, and the end of the study period in 2023. To summarize the type and extent of sedation use whilst in critical care, the use rate and duration in days were reported, broken down by sedative agent (propofol, dexmedetomidine, lorazepam, midazolam, ketamine, fentanyl, and hydromorphone).

Primary and Secondary Outcomes

The primary outcome, ICU-free days, was calculated by subtracting the total time spent in any ICU from the total hospital length of stay using documented admission/discharge dates and times; for patients who received care in multiple ICUs or were admitted and discharged from intensive care multiple times within the same hospital encounter, the times were collated to determine total time spent in ICU.

Secondary continuous outcomes included endotracheal intubation-, mechanical ventilation- (MV), and inpatient antimicrobial-free days. Available documentation of start/stop dates and times was used to determine total durations of endotracheal intubation and mechanical ventilation, then subtracted from total hospital length of stay, consistent with the approach used to calculate total ICU-free days. Duration of antimicrobial therapy was defined by time elapsed from the first to last dose of antimicrobial medication given during hospitalization; likewise, the duration of antimicrobial therapy was subtracted from total hospital length of stay to determine antimicrobial-free days. Frequency, degree, and duration of infusion vasopressor use were also reported; the number of different vasopressor infusion medications used during hospitalization was reported in a stratified manner (0, 1-2, or 3-4 medications administered) to reflect extent of use. Frequency of use and duration in days were reported for each specific vasopressor medication administered (including norepinephrine, epinephrine, phenylephrine, and vasopressin). Categorical outcomes included rates of mortality (in-hospital or within 30 days of discharge date) and sepsis recurrence within six months of discharge from the index sepsis encounter. Time to sepsis recurrence, presented in days, was determined for all cases of sepsis recurrence within six months. Time to infection clearance from the blood was additionally reported, as available and as applicable. Infection clearance was defined as the date and time of the first negative blood culture without any subsequent positive blood cultures; thus, time to infection-blood clearance was only reported in cases where a positive blood culture was identified followed by repeat culturing. Within the propofol group only, duration and overall infusion rate administered during hospitalization were determined and reported in hours and milligrams per hour, respectively. These data were compared among patients who expired versus survived and among patients with versus without six-month sepsis recurrence.

Statistical analysis

Categorical data were described with counts and frequencies and compared with Pearson Chi-Square tests or Fisher's Exact tests when cell counts were low.

Continuous, non-normally distributed data were depicted using median and interquartile range (IQR). For independent data comparisons, Mann-Whitney U tests were used; for paired data, Wilcoxon Signed Rank tests were performed. When the number of matched pairs was low, independent group comparison was retained with Mann-Whitney U tests.

To conduct the propensity score matching (PSM), the predictive probability of assigning patients to propofol was first generated using standard binary logistic regression without replacement using a caliper width of 0.025 on the propensity-score scale. Covariates in the model included sepsis versus septic shock diagnosis, demographic factors (age, sex, BMI), and CCI score at the time of hospital admission, matched in the order written. The probabilities were matched using greedy algorithms to identify 1:1 comparable propofol versus non-propofol subpopulations to scrutinize the effect of propofol in the SAS OnDemand platform (SAS OnDemand for Academics, SAS Institute, Cary, NC, USA). Visual inspection of propensity score histograms confirmed adequate common support across study groups (see Appendix B). A two-sided alpha level of 0.05 was used for all hypothesis testing; results with a p-value <0.05 were considered statistically significant. All statistical procedures were carried out using IBM SPSS Statistics for Windows, version 29 (IBM Corp., Armonk, NY, USA).

## Results

Primary analysis

A total of 615 patients during the study period were identified, comprising 511 (83.1%) propofol-sedated patients and 104 (16.9%) non-propofol-sedated patients (Figure 1).

**Figure 1:**
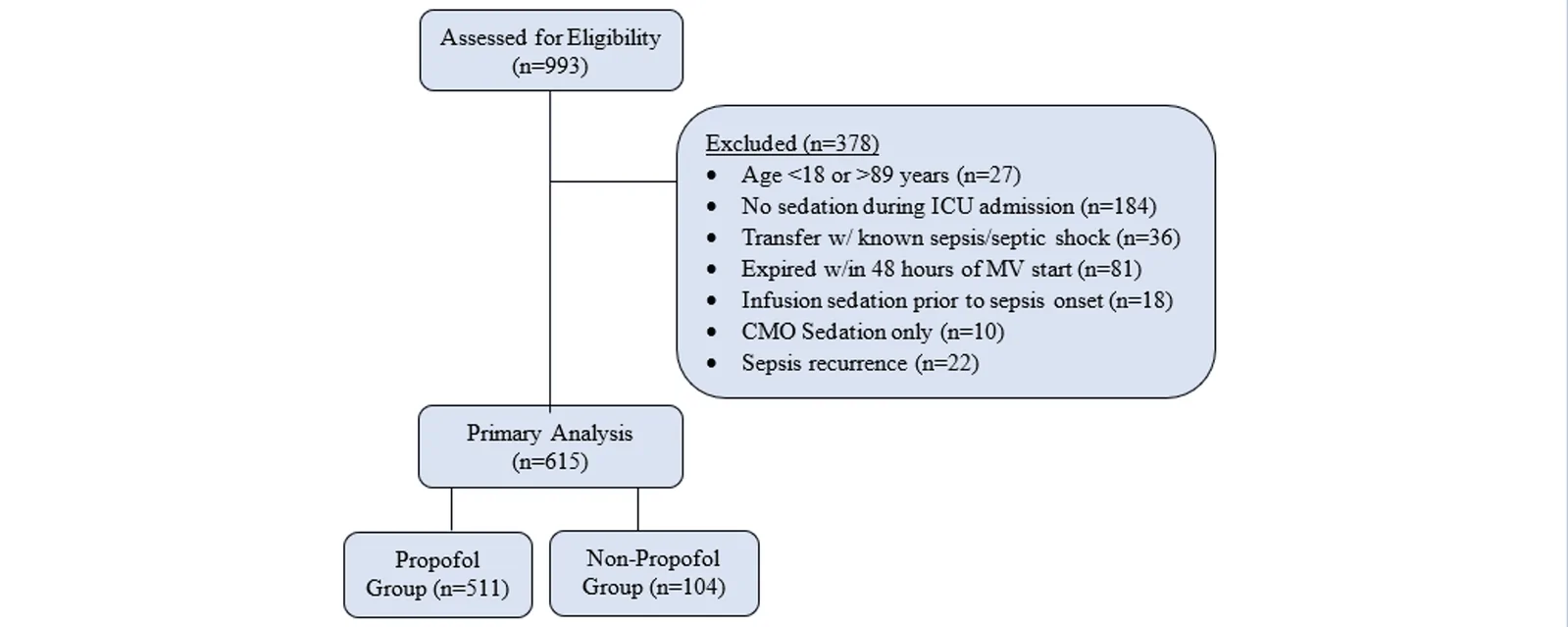
Participant Flow Diagram MV, Mechanical Ventilation; CMO, Comfort Measures Only

Demographic and Baseline Characteristics

Comparison of demographic and baseline characteristics between groups yielded several statistically significant differences (Table 1). Patients in the non-propofol group were older, on average, than those in the propofol group (66 (58-75) vs. 62 (50-72) years, p=0.021). The frequency of patients receiving non-propofol sedation was greater between 2017 and 2019 (non-propofol, 64.4%), whereas between 2020 and 2022, propofol sedation was predominant (propofol, 55.8%) (p<0.001). Patients treated for septic shock comprised most of the dataset (n=498, 81.0%). Though septic shock diagnosis was more common in the non-propofol group, this difference was not statistically significant (91 (87.5%) vs. 407 (79.6%), p=0.063). The distribution of identified infection sources differed significantly between the non-propofol and propofol groups (p<0.001).

**Table 1:** Demographics and Baseline Health Characteristics: Original Dataset *Frequencies are calculated based on encounters between 2020-2022: non-propofol group, n=37; propofol group, n=285 IQR, Interquartile Range; BMI, Body Mass Index; CCI, Charlson Comorbidity Index; MDR, Multi-Drug Resistant; CNS, Central Nervous System; COVID-19, Coronavirus Disease of 2019

Variables	Categories	Non-Propofol (n=104)	Propofol (n=511)	Standardized Mean Difference	p-value	Test Statistic Value	Statistical Test
Age, median (IQR)		66 (58-75)	62 (50-72)	0.265	0.021	30383	Mann-Whitney U
BMI, median (IQR)		27.3 (22.4-32.2)	27.5 (22.7-33.5)	-0.133	0.373	25051	Mann-Whitney U
Sex, n (%)	Female	41 (39.4)	191 (37.4)	0.041	0.695	0.154	Chi-Squared
	Male	63 (60.6)	320 (62.6)				
Ethnicity, n (%)	Hispanic or Latino	13 (12.5)	88 (17.2)	-0.132	0.423	1.722	Chi-Squared
	Not Hispanic or Latino	89 (85.6)	409 (80.0)	0.149			
	Unknown	2 (1.9)	14 (2.7)	-0.053			
Race, n (%)	White or Caucasian	75 (72.1)	334 (65.4)	0.145	0.605	1.844	Chi-Squared
	Black or African American	9 (8.7)	58 (11.4)	-0.090			
	Other	18 (17.3)	105 (20.5)	-0.082			
	Unknown	2 (1.9)	14 (2.7)	-0.053			
CCI, median (IQR)		4 (2-6)	3 (2-5)	0.172	0.133	29031	Mann-Whitney U
Admit Year, n (%)	2017-2019	67 (64.4)	226 (44.2)	0.414	<0.001	14.130	Chi-Squared
	2020-2022	37 (35.6)	285 (55.8)				
Diagnosis, n (%)	Sepsis	13 (12.5)	104 (20.4)	-0.214	0.063	3.459	Chi-Squared
	Septic Shock	91 (87.5)	407 (79.6)				
Positive Blood Culture, n (%)	Gram Positive	19 (18.3)	97 (19.0)	-0.018	0.754	1.193	Chi-Squared
	Gram Negative	18 (17.3)	70 (13.7)	0.099			
	Fungal or MDR	7 (6.7)	44 (8.6)	-0.072			
	Unknown	60 (57.7)	300 (58.7)	-0.020			
Infection Source, n (%)	Respiratory	28 (26.9)	130 (25.4)	0.034	<0.001	25.346	Chi-Squared
	Urinary Tract	34 (32.7)	88 (17.2)	0.364			
	Gastrointestinal	7 (6.7)	14 (2.7)	0.190			
	Skin & Soft Tissue	4 (3.8)	64 (12.5)	-0.322			
	Direct	2 (1.9)	13 (2.5)	-0.041			
	Endocarditis	0 (0.0)	11 (2.2)	-0.212			
	Osteomyelitis	0 (0.0)	2 (0.4)	-0.090			
	CNS	0 (0.0)	1 (0.2)	-0.063			
	Unknown	29 (27.9)	188 (36.8)	-0.191			
Pharmaceutical Class, n (%)	Antifungals	21 (20.2)	67 (13.1)	0.191	0.060	3.533	Chi-Squared
	Beta-Lactams	94 (90.4)	467 (91.4)	-0.035	0.741	0.109	Chi-Squared
	Fluoroquinolones	14 (13.5)	95 (18.6)	-0.139	0.212	1.559	Chi-Squared
	Macrolide Antibiotics	11 (10.6)	66 (12.9)	-0.071	0.511	0.432	Chi-Squared
	Miscellaneous Anti-infectives	102 (98.1)	486 (95.1)	0.166	0.178	1.815	Chi-Squared
Lipid Emulsion, n (%)		8 (7.7)	27 (5.3)	0.097	0.334	0.934	Chi-Squared
Positive COVID test*, n (%)		1 (2.4)	24 (8.4)	-0.268	0.333	-	Fisher’s Exact
Suspected or known COVID-19 diagnosis*, n (%)		3 (8.1)	41 (14.4)	-0.200	0.445	-	Fisher’s Exact

There were no differences in frequency or duration of non-propofol sedation with the exception of fentanyl (Table 2); the non-propofol group received fentanyl sedation more commonly (84 (80.8%) vs. 234 (45.8%), p<0.001), though median duration of fentanyl use was longer in the propofol group (1.9 (0.9-4.2) vs. 1.5 (0.4-2.8) days, p=0.012).

**Table 2:** Sedation Characteristics: Original Dataset IQR, Interquartile Range

Medication	Variable	Non-Propofol (n=104)	Propofol (n=511)	Standardized Mean Difference	p-value	Test Statistic Value	Statistical Test
Sedative Agents							
Propofol	Duration, days, median (IQR)	-	1.0 (0.4-2.4)	-			
Dexmedetomidine	n (%)	32 (30.8)	174 (34.1)	-0.071	0.518	0.418	Chi-Squared
	Duration, days, median (IQR)	1.0 (0.5-2.4)	1.4 (0.4-3.6)	-0.181	0.499	2574	Mann-Whitney U
Lorazepam	n (%)	13 (12.5)	68 (13.3)	-0.024	0.824	0.049	Chi-Squared
	Duration, days, median (IQR)	3.0 (1.7-7.7)	3.2 (1.5-5.7)	0.174	0.762	466	Mann-Whitney U
Midazolam	n (%)	11 (10.6)	45 (8.8)	0.061	0.567	0.327	Chi-Squared
	Duration, days, median (IQR)	0.5 (0.2-2.0)	0.8 (0.4-1.4)	-0.252	0.788	234	Mann-Whitney U
Analgo-sedation Agents							
Ketamine	n (%)	6 (5.8)	38 (7.4)	-0.064	0.548	0.362	Chi-Squared
	Duration, days, median (IQR)	4.7 (1.4-10.1)	3.4 (0.9-5.7)	0.310	0.584	131	Mann-Whitney U
Fentanyl	n (%)	84 (80.8)	234 (45.8)	0.779	<0.001	42.335	Chi-Squared
	Duration, days, median (IQR)	1.5 (0.4-2.8)	1.9 (0.9-4.2)	-0.297	0.012	3431	Mann-Whitney U
Hydromorphone	n (%)	26 (25.0)	142 (27.8)	-0.064	0.561	0.339	Chi-Squared
	Duration, days, median (IQR)	2.2 (0.8-5.1)	2.3 (0.9-5.9)	0.232	0.939	1864	Mann-Whitney U

Primary and Secondary Outcomes

Median ICU-free days were similar between non-propofol and propofol-sedated groups (6.3 (2.1-14.7) vs. 6.8 (3.3-13.3) days, p=0.303) (Table 3). Likewise, there were no statistically significant differences in endotracheal intubation-, mechanical ventilation-, or inpatient antimicrobial-free days. The proportion of patients requiring no pressor infusions was significantly greater in the propofol group (102 (20.0%) vs. 9 (8.7%), p=0.006); conversely, the proportion of patients requiring multiple vasopressor infusions was significantly greater in the non-propofol group (3 or 4 infusions, 32 (30.8%) vs. 110 (21.5%), p=0.041). Frequencies of norepinephrine, phenylephrine, and vasopressin infusion use were greater in the non-propofol group (94 (90.4%) vs. 370 (72.4%), p<0.001; 46 (44.2%) vs. 173 (33.9%), p=0.044; 46 (44.2%) vs. 168 (32.9%), p=0.027, respectively). However, there were no differences in infusion duration of these vasopressor medications. The combined hospital or 30-day mortality rate was markedly higher in the non-propofol group (45 (43.3%) vs. 165 (32.3%), p=0.031). No differences in six-month sepsis recurrence rate, time to sepsis recurrence, or time to infection clearance from blood were found between groups. 

**Table 3:** Clinical Outcomes: Original Dataset *Recurrence measured only among patients who did not pass away during index sepsis encounter (non-propofol, n=59; propofol, n=346) **Time to infection clearance from blood calculated only among patients with an available positive blood culture and negative culture before hospital discharge (non-propofol, n=40; propofol, n=193) IQR, Interquartile Range; MV, Mechanical Ventilation; ICU, Intensive Care Unit

Variables		Categories	Non-Propofol (n=104)	Propofol (n=511)	Median Difference	p-value	Test Statistic Value	Effect Size	95% CI	Statistical Test
ICU-free days, median (IQR)			6.3 (2.1-14.7)	6.8 (3.3-13.3)	0.5	0.303	24771	0.042	-0.022, 0.106	Mann-Whitney U
Intubation-free days, median (IQR)			9.2 (4.5-19.0)	9.6 (5.3-18.9)	0.4	0.624	25762	0.020	-0.010, 0.050	Mann-Whitney U
MV-free days, median (IQR)			8.8 (4.5-18.1)	8.7 (4.9-16.3)	0.1	0.891	26800	0.006	-0.003, 0.014	Mann-Whitney U
Antimicrobial-free days, median (IQR)			8.0 (4.3-13.1)	7.3 (3.5-14.3)	0.7	0.717	27171	0.015	-0.008, 0.038	Mann-Whitney U
Vasopressor Infusion Count, n (%)		0	9 (8.7)	102 (20.0)	-	0.006	7.469	0.110	0.031, 0.187	Chi-Squared
		1 or 2	63 (60.6)	299 (58.5)	-	0.697	0.152	0.016	-0.063, 0.095	Chi-Squared
		3 or 4	32 (30.8)	110 (21.5)	-	0.041	4.157	0.082	0.003, 0.160	Chi-Squared
Vasopressor Medication Use & Duration	Norepinephrine	n (%)	94 (90.4)	370 (72.4)	-	<0.001	15.076	0.157	0.079, 0.233	Chi-Squared
		Starting Dose, mL/h, median (IQR)	11.3 (7.5-21.6)	9.4 (7.5-26.3)	1.9	0.199	18871	0.060	-0.031, 0.150	Mann-Whitney U
		Duration, Days, median (IQR)	2.0 (1.0-3.2)	1.5 (0.7-3.2)	0.5	0.089	19368	0.079	-0.035, 0.193	Mann-Whitney U
	Epinephrine	n (%)	10 (9.6)	59 (11.5)	-	0.570	0.323	0.023	-0.056, 0.102	Chi-Squared
		Duration, Days, median (IQR)	0.5 (0.1-1.7)	0.7 (0.1-1.7)	0.2	0.720	274	0.043	-0.028, 0.114	Mann-Whitney U
	Phenylephrine	n (%)	46 (44.2)	173 (33.9)	-	0.044	4.057	0.081	0.002, 0.159	Chi-Squared
		Duration Days, median (IQR)	0.6 (0.3-1.2)	0.6 (0.2-1.5)	0.0	0.491	3716	0.047	-0.019, 0.113	Mann-Whitney U
	Vasopressin	n (%)	46 (44.2)	168 (32.9)	-	0.027	4.910	0.089	0.010, 0.167	Chi-Squared
		Duration Days, median (IQR)	2.0 (1.1-3.8)	1.6 (0.6-3.4)	0.4	0.118	4446	0.110	-0.041, 0.261	Mann-Whitney U
Hospital & 30-Day Mortality, n (%)			45 (43.3)	165 (32.3)	-	0.031	4.633	0.087	0.008, 0.165	Chi-Squared
Sepsis Recurrence within Six Months*, n (%)			8 (13.6)	70 (20.2)	-	0.230	1.443	0.060	-0.038, 0.157	Chi-Squared
Time to Recurrence*, days, median (IQR)			67 (39-84)	56 (21-126)	11	0.876	290	0.018	-0.016, 0.052	Mann-Whitney U
Positive Blood Culture with Repeat Cultures, n (%)			40 (38.5)	193 (37.8)	-	0.894	0.018	0.005	-0.124, 0.133	Chi-Squared
Time to Infection Clearance from Blood**, days, median (IQR)			2.3 (1.3-9.1)	2.9 (1.3-5.9)	0.6	0.909	3905	0.008	-0.004, 0.020	Mann-Whitney U

Mann-Whitney U testing revealed no statistically significant differences in duration of propofol sedation among patients who expired during or within 30 days of discharge from sepsis hospitalization versus patients who survived (Table 4). The propofol infusion rate was significantly higher among patients who survived versus expired, however (120.3 (81.4-182.7) vs. 105.0 (63.8-161.9) mg/hour, p=0.008). Comparing patients with versus without sepsis recurrence, there were no differences in duration of propofol sedation or infusion rate.

**Table 4:** Association of Propofol Infusion Duration and Magnitude With Incidence of Mortality and Sepsis Recurrence *Recurrence measured only among patients who did not pass away during index sepsis encounter (propofol, n=346) IQR, Interquartile Range

Variables	Outcome		Median Difference	p-value	Test Statistic	Effect Size	95% CI	Statistical Test
-	Hospital or 30-Day Mortality		-					
	yes	no						
Propofol Sedation, hours, median (IQR)	26.0 (9.0-66.0)	22.0 (9.0-50.8)	4.0	0.244	26727	0.052	-0.012, 0.116	Mann-Whitney U
Propofol Infusion Rate, mg/hour, median (IQR)	105.0 (63.8-161.9)	120.3 (81.4-182.7)	15.3	0.008	32661	0.120	-0.024, 0.264	Mann-Whitney U
-	Sepsis Recurrence*		-					
	yes	no						
Propofol Sedation, hours, median (IQR)	22.0 (9.0-53.0)	21.0 (8.0-50.0)	1.0	0.593	14301	0.028	-0.008, 0.064	Mann-Whitney U
Propofol Infusion Rate, mg/hour, median (IQR)	117.7 (70.9-189.4)	119.0 (77.4-179.5)	1.3	0.822	13600	0.012	-0.003, 0.027	Mann-Whitney U

PSM analysis

Of the comparable subpopulations (n=104 each) identified using PSM, almost all patient demographics and baseline clinical characteristics were similar between groups, with the exception of non-propofol versus propofol sedation use in 2017-2019 versus 2020-2022 (Table 5). The frequency of non-propofol sedation remained greater in 2017 to 2019 (64.4%), while propofol sedation remained more frequent between 2020 and 2022 (58.7%) (p<0.001). The distribution of identifiable infection sources also remained significantly different between groups (p=0.041).

**Table 5:** Demographics and Baseline Health Characteristics: PSM Data *Matched pair counts less than 104 reflect ties **Frequencies are calculated based on encounters between 2020-2022: non-propofol group, n=37; propofol group, n=61 IQR, Interquartile Range; BMI, Body Mass Index; CCI, Charlson Comorbidity Index; MDR, Multi-Drug Resistant; COVID-19, Coronavirus Disease of 2019; PSM, Propensity Score Matching

Variables	Categories		Non-Propofol (n=104)	Propofol (n=104)	Standardized Mean Difference	p-value	Test Statistic Value	Matched Pairs* n (%)	Statistical Test
Age, median (IQR)			66 (58-75)	67 (58-75)	-0.062	0.468	2361	101	Wilcoxon Signed-Rank Test
BMI, median (IQR)			27.3 (22.4-32.2)	27.9 (23.1-32.0)	0.026	0.899	2691	104	Wilcoxon Signed-Rank Test
Sex, n (%)	Female		41 (39.4)	37 (35.6)	0.079	0.567	0.328	-	Chi-Squared
	Male		63 (60.6)	67 (64.4)					
Ethnicity, n (%)	Hispanic or Latino		13 (12.5)	17 (16.3)	-0.108	0.632	0.918	-	Chi-Squared
	Not Hispanic or Latino		89 (85.6)	86 (82.7)	0.079				
	Unknown		2 (1.9)	1 (1.0)	0.075				
Race, n (%)	White or Caucasian		75 (72.1)	68 (65.4)	0.145	0.603	1.856	-	Chi-Squared
	Black or African American		9 (8.7)	8 (7.7)	0.036				
	Other		18 (17.3)	26 (25.0)	-0.189				
	Unknown		2 (1.9)	2 (1.9)	0				
CCI, median (IQR)			4 (2-6)	3 (2-5)	0.165	0.322	1681	87	Wilcoxon Signed-Rank Test
Admit Year, n (%)		2017-2019	67 (64.4)	43 (41.3)	0.476	<0.001	11.114	-	Chi-Squared
		2020-2022	37 (35.6)	61 (58.7)					
Diagnosis, n (%)		Sepsis	13 (12.5)	13 (12.5)	0	1.000	0	-	Chi-Squared
		Septic Shock	91 (87.5)	91 (87.5)					
Positive Blood Culture, n (%)		Gram Positive	19 (18.3)	27 (26.0)	-0.186	0.429	2.765	-	Chi-Squared
		Gram Negative	18 (17.3)	15 (14.4)	0.079				
		Fungal or MDR	7 (6.7)	10 (9.6)	-0.106				
		Unknown	60 (57.7)	52 (50.0)	0.155				
Infection Source, n (%)		Respiratory	28 (26.9)	29 (27.9)	-0.022	0.041	14.611	-	Chi-Squared
		Urinary Tract	34 (32.7)	17 (16.3)	0.388				
		Gastrointestinal	7 (6.7)	2 (1.9)	0.238				
		Skin & Soft Tissue	4 (3.8)	6 (5.8)	-0.094				
		Direct	2 (1.9)	4 (3.8)	-0.114				
		Endocarditis	0 (0.0)	1 (1.0)	-0.142				
		Osteomyelitis	0 (0.0)	1 (1.0)	-0.142				
		Unknown	29 (27.9)	44 (42.3)	-0.305				
Pharmaceutical Class, n (%)		Antifungals	21 (20.2)	15 (14.4)	0.154	0.271	1.209	-	Chi-Squared
		Beta-Lactams	94 (90.4)	94 (90.4)	0	1.000	0	-	Chi-Squared
		Fluoroquinolones	14 (13.5)	18 (17.3)	-0.105	0.442	0.591	-	Chi-Squared
		Macrolide Antibiotics	11 (10.6)	17 (16.3)	-0.168	0.223	1.486	-	Chi-Squared
		Miscellaneous Anti-infectives	102 (98.1)	0	0	1.000	0	-	Chi-Squared
Lipid Emulsion, n (%)			8 (7.7)	10 (9.6)	-0.068	0.622	0.243	-	Chi-Squared
Positive COVID test**, n (%)			1 (2.4)	5 (8.2)	-0.261	0.404	-	-	Fisher’s Exact
Suspected or known COVID-19 diagnosis**, n (%)			3 (8.1)	8 (13.1)	-0.163	0.527	-	-	Fisher’s Exact

Frequency of fentanyl sedation remained significantly greater in the non-propofol group (84 (80.8%) vs. 49 (47.1%), p<0.001), and median duration remained longer in the propofol group (2.2 (1.1-4.3) vs. 1.5 (0.4-2.8) days, p=0.006) (Table 6). The use and duration of other sedatives remained similar between groups.

**Table 6:** Sedation Characteristics: PSM Dataset IQR, Interquartile Range; PSM, Propensity Score Matching

Medication	Variable	Non-Propofol (n=104)	Propofol (n=104)	Standardized Mean Difference	p-value	Test Statistic Value	Statistical Test
Propofol	Duration, days, median (IQR)	-	1.2 (0.5-2.8)	-			-
Dexmedetomidine	n (%)	32 (30.8)	39 (37.5)	-0.142	0.306	1.048	Chi-Squared
	Duration, days, median (IQR)	1.0 (0.5-2.4)	1.0 (0.4-3.6)	-0.205	0.873	619	Mann-Whitney U
Lorazepam	n (%)	13 (12.5)	11 (10.6)	0.059	0.664	0.188	Chi-Squared
	Duration, days, median (IQR)	3.0 (1.7-7.7)	4.0 (2.8-5.3)	0.250	0.910	69	Mann-Whitney U
Midazolam	n (%)	11 (10.6)	9 (8.7)	0.064	0.638	0.221	Chi-Squared
	Duration, days, median (IQR)	0.5 (0.2-2.0)	0.8 (0.4-1.2)	-0.282	0.970	48.5	Mann-Whitney U
Ketamine	n (%)	6 (5.8)	8 (7.7)	-0.076	0.580	0.306	Chi-Squared
	Duration, days, median (IQR)	4.6 (1.4-10.1)	4.3 (2.4-5.6)	0.193	1.000	24	Mann-Whitney U
Fentanyl	n (%)	84 (80.8)	49 (47.1)	0.750	<0.001	25.544	Chi-Squared
	Duration, days, median (IQR)	1.5 (0.4-2.8)	2.2 (1.1-4.3)	-0.413	0.006	1473	Mann-Whitney U
Hydromorphone	n (%)	26 (25.0)	30 (28.8)	-0.086	0.532	0.391	Chi-Squared
	Duration, days, median (IQR)	2.2 (0.8-5.1)	1.5 (0.2-5.3)	0.291	0.316	452	Mann-Whitney U

ICU-, endotracheal intubation-, mechanical ventilation-, and antimicrobial-free days remained similar between groups, consistent with results of the primary analysis (Table 7). Extent of infusion vasopressor requirement and frequencies of specific vasopressor medications used became similar between groups. Despite similar vasopressin use rates, the median duration of vasopressin infusion was longer in the non-propofol group (2.0 (1.1-3.8) vs. 0.9 (0.6-3.0) days, p=0.013). Combined hospital or 30-day mortality incidence was similar between groups, without differences in rates of sepsis recurrence, time to sepsis recurrence, or time to infection clearance from blood.

**Table 7:** Clinical Outcomes: PSM Data *Matched pair counts less than 104 reflect ties **Recurrence measured only among patients who did not pass away during index sepsis encounter (non-propofol, n=59; propofol, n=62) ***Time to infection clearance from blood calculated only among patients with an available positive blood culture and negative culture before hospital discharge (non-propofol, n=40; propofol, n=36) IQR, Interquartile Range; MV, Mechanical Ventilation; ICU, Intensive Care Unit; PSM, Propensity Score Matching

Variables		Categories	Non-Propofol (n=104)	Propofol (n=104)	Median Difference	p-value	Test Statistic Value	Effect Size	95% CI	Number Matched Pairs*	Statistical Test
ICU-free days, median (IQR)			6.3 (2.1-14.7)	6.7 (2.9-12.0)	0.4	0.896	2587	0.013	0.002, 0.024	102	Wilcoxon Signed-Rank Test
Intubation-free days, median (IQR)			9.2 (4.5-19.0)	10.3 (4.7-19.0)	1.1	0.735	2575	0.033	0.012, 0.066	104	Wilcoxon Signed-Rank Test
MV-free days, median (IQR)			8.8 (4.5-18.1)	9.4 (4.5-16.6)	0.2	0.694	2608	0.039	0.007, 0.071	104	Wilcoxon Signed-Rank Test
Antimicrobial-free days, median (IQR)			8.0 (4.3-13.1)	8.4 (3.5-14.7)	0.4	0.820	2748	0.022	0.004, 0.040	104	Wilcoxon Signed-Rank Test
Vasopressor Infusion Count, n (%)		0	9 (8.7)	9 (8.7)	-	1.000	0	0	-0.079, 0.079	-	Chi-Squared
		1 or 2	63 (60.6)	61 (58.7)	-	0.777	0.080	0.020	-0.059, 0.099	-	Chi-Squared
		3 or 4	32 (30.8)	34 (32.7)	-	0.767	0.089	0.021	-0.058, 0.100	-	Chi-Squared
Vasopressor Medication Use & Duration	Norepinephrine	n (%)	94 (90.4)	87 (83.7)	-	0.149	2.086	0.100	0.021, 0.178	-	Chi-Squared
		Starting Dose, mL/h, median (IQR)	13.2 (7.5-24.4)	9.4 (5.4-28.1)	3.8	0.455	1181	0.088	-0.147, 0.313	72	
		Duration, Days, median (IQR)	2.0 (1.0-3.3)	1.7 (0.7-3.1)	0.3	0.200	1318	0.144	-0.012, 0.294	79	Wilcoxon Signed-Rank Test
	Epinephrine	n (%)	10 (9.6)	7 (6.7)	-	0.448	0.577	0.053	-0.026, 0.132	-	Chi-Squared
		Duration, Days, median (IQR)	0.5 (0.1-1.7)	1.1 (0.9-1.9)	0.6	0.171	21	0.330	-0.180, 0.700	-	Mann-Whitney U
	Phenylephrine	n (%)	46 (44.2)	38 (36.5)	-	0.258	1.278	0.078	-0.001, 0.156	-	Chi-Squared
		Duration, Days, median (IQR)	0.6 (0.3-1.2)	0.6 (0.2-1.4)	0.0	0.777	842	0.031	-0.185, 0.244	-	Mann-Whitney U
	Vasopressin	n (%)	46 (44.2)	41 (39.4)	-	0.482	0.049	0.049	-0.030, 0.128	-	Chi-Squared
		Duration, Days, median (IQR)	2.0 (1.1-3.8)	0.9 (0.6-3.0)	1.1	0.013	1210	0.270	0.063, 0.455	-	Mann-Whitney U
Hospital & 30-Day Mortality, n (%)			45 (43.3)	42 (40.4)	-	0.673	0.178	0.029	-0.050, 0.108	-	Chi-Squared
Sepsis Recurrence within Six Months**, n (%)			8 (13.6)	11 (17.7)	-	0.527	0.400	0.058	-0.122, 0.234	-	Chi-Squared
Time to Recurrence**, days, median (IQR)			67 (39-84)	55 (24-106)	12	0.967	43	0.010	-0.446, 0.462	-	Mann-Whitney U
Positive Blood Culture with Repeat Cultures, n (%)			40 (38.5)	36 (34.6)	-	0.565	0.332	0.040	-0.187, 0.263	-	Chi-Squared
Time to Infection Clearance from Blood***, days, median (IQR)			2.3 (1.3-9.1)	2.1 (1.3-3.8)	0.2	0.315	817	0.120	-0.108, 0.336	-	Mann-Whitney U

## Discussion

Propofol infusions were numerically less common among older patients and those with septic shock versus isolated sepsis diagnosis. Fentanyl was the most common means of analgo-sedation in the non-propofol group; this group also demonstrated increased vasopressor support requirements compared to the propofol group. After PSM for confounding factors, propofol sedation did not reduce ICU-, endotracheal intubation- or mechanical ventilation-free days; likewise, propofol sedation was not associated with fewer antimicrobial-free days or differences in time to infection-blood clearance, six-month sepsis recurrence, or mortality rates.

Most patients received propofol (n=511, 83.1%) versus non-propofol (n=104, 16.9%) sedation. Given propofol’s rapid onset of action and short context-sensitive half-life, these findings were not unexpected. Current practice guidelines support light sedation strategies during mechanical ventilation, often using propofol (or dexmedetomidine) as adjuncts within an analgesia-first approach. However, propofol infusions are sometimes avoided in patients with septic shock because of their potential to induce or worsen hypotension. In this context, the higher incidence of septic shock diagnoses and greater need for vasopressor support observed in the non-propofol group were clinically reasonable. The risk of septic shock and the burden of chronic comorbidities increase with advancing age, which may explain the older age and higher comorbidity burden observed in the non-propofol group prior to propensity score matching. Progression to septic shock, advanced age, and greater comorbidity burden are well-established predictors of mortality following sepsis. Consistent with this, our data demonstrate significantly higher mortality in the non-propofol group, reflecting the elevated baseline risk within this subset. Notably, no significant differences were observed in other clinical outcomes, including ICU-, endotracheal intubation- or mechanical ventilation-free days. This may be attributable to the heterogeneous composition of the cohort, which likely included both critically ill patients who died early during hospitalization and patients with prolonged, complex ICU courses related to shock and multiorgan dysfunction requiring procedural interventions and extended supportive care.

Following one-to-one PSM, accounting for sepsis versus septic shock diagnosis, age, sex, BMI, and CCI score, the groups were more comparable without significant differences in nearly all baseline characteristics, confirming matching success. Frequency of non-propofol versus propofol sedation remained significantly different before versus after 2020. Non-propofol sedation was used more frequently between 2017 and 2019, whereas propofol sedation predominated between 2020 and 2022. This may, in part, be related to reaffirmed Pain, Agitation/sedation, Delirium, Immobility (rehabilitation/mobilization), and Sleep (disruption) (PADIS) clinical guidelines released later in 2018, which recommended preferred use of propofol or dexmedetomidine over benzodiazepine-mediated sedation [18]. Moreover, the frequency of fentanyl sedation remained significantly higher in the non-propofol group after PSM. The 2018 PADIS guidelines additionally recommended an analgesia-first approach to sedation, wherein pain is targeted first with intravenous opioids before addition of any sedative [18]. Since fentanyl functions as both an analgesic and sedative, it is reasonable that fentanyl sedation was used as first-line in the absence of adjunct propofol sedation. The significantly longer duration of fentanyl sedation seen in the propofol group is less easily explained, requiring consideration of multiple influencing factors including infusion timing, concurrent or adjacent use of other sedatives, and sedative-specific, context-sensitive half-times. Nonetheless, it is unlikely the differences in fentanyl sedation had any substantial impacts on the outcomes measured. Indeed, a recent systematic review and meta-analysis reported that fentanyl sedation in mechanically ventilated patients was not associated with prolonged mechanical ventilation or ICU length of stay, nor was its use associated with increased delirium or mortality rates [19].

Post-PSM data revealed similar combined hospital or 30-day mortality rates between groups, affirming confounding influence at baseline rather than an intervention-related difference. Consistent with this finding, existing research indicates no differences in mortality rates when propofol versus alternative sedation is used [20-22]. ICU-, endotracheal intubation-, and mechanical ventilation-free days remained similar between groups after PSM adjustment. A similarly designed retrospective, propensity-matched pilot study by Marler et al. compared propofol versus non-propofol sedation in mechanically ventilated patients with sepsis and, likewise, reported no differences in ICU length of stay or duration of mechanical ventilation [22]. Two randomized controlled trials evaluated propofol versus dexmedetomidine sedation among septic and non-septic, critically ill patients and also reported no differences in median duration of mechanical ventilation or ventilator-free days [20,21].

Post-PSM antimicrobial-free days were also similar between groups. Antibiotic start and discontinuation are contingent upon multiple factors, including the patient’s clinical state, infection clearance from blood and adequate source control, and planned duration of empirical therapy, among others [23]. As such, this finding was not surprising.

Our post-PSM adjusted data additionally indicated comparable vasopressor support requirements, with the exception of longer vasopressin duration in the non-propofol group. Marler et al. did not identify any significant difference in vasopressin use or duration; they did, however, report enhanced epinephrine requirements in the non-propofol versus propofol group following PSM [22]. Between the 2016 and 2021 updates of the Surviving Sepsis Campaign (SSC) guidelines, the recommendation for second-line vasopressor therapy evolved from the use of either vasopressin or epinephrine to a preference for vasopressin over epinephrine as the second-line agent to achieve target mean arterial pressure [23,24]. Thus, the discrepancy in vasopressin duration from the Marler et al. report is likely due to changes in clinical practice [22]. Enhanced pressor use in the non-propofol group seen in both retrospective reports may be due to provider bias to avoid propofol when patients demonstrate prolonged, refractory hemodynamic instability.

Previous preclinical research has associated propofol exposure with increased bacterial burden, dissemination, and immunosuppression [3-9]; however, translational assessment of these parameters in the clinical setting remains a challenge. In cases of suspected or known sepsis, it is standard clinical practice to collect a blood culture set to assess for bacterial presence prior to antibiotic initiation. Repeat cultures are performed when there is a lack of clinical response within two to three days of antimicrobial use, clinical deterioration is noted, or when initial blood cultures are positive for high-risk pathogens such as *Staphylococcus aureus* or *Candida* species. Repeat culturing is also used when a patient initially stabilizes, then later develops new signs of sepsis during hospitalization. We, therefore, used time to blood-infection clearance as an indirect proxy of bacterial burden and response to antibiotics. We found that rates of positive blood culture followed by repeat culturing were similar between groups without a significant difference in time to infection-blood clearance. Likewise, six-month sepsis recurrence and time to sepsis recurrence were similar between groups. Taken together, exposure to propofol sedation was not associated with prolonged time to infection-blood clearance or increased risk of sepsis recurrence in the setting of sepsis with mechanical ventilation requirement.

This study has limitations to consider. This retrospective analysis relied on available clinical documentation; any lack or error in documentation could have influenced our findings. For example, recurrence and mortality rates were reported based on available data in our healthcare system; hospitalizations for sepsis elsewhere and non-reported mortality were not considered in this analysis and reflect incomplete follow-up. Grouping by any propofol use might have been too simple, considering that, in some circumstances, propofol sedation was only used temporarily before transitioning to other sedatives. Moreover, the duration of propofol exposure was relatively short in our cohort, potentially limiting the study's capacity to assess dose- and duration-dependent, clinically translational immunological effects. Patients who expired within 48 hours of mechanical ventilation start were excluded from analysis, which reduces the generalizability of our findings and capacity to detect harm related to propofol exposure. Previous studies have used the Acute Physiology and Chronic Health Evaluation II (APACHE II) assessment rather than or alongside CCI to summarize baseline health and mortality risk [25]. APACHE II model comprises an array of physiological indicators, while CCI comprehensively summarizes existing chronic comorbidity burden. Though APACHE II is generally preferred for acute mortality risk assessment in critical care settings, CCI has been shown to perform at a similar level to physiology-based models when combined with age, sex, and MV status [26]. Intensivists may have avoided propofol sedation in more critically ill patients presenting with shock due to its known hypotensive effects. To account for confounding influence due to shock status as well as acute and chronic illness severity, both CCI and sepsis/septic shock status were accounted for in the propensity matching technique. PSM-paired analysis was not performed for some outcomes such as duration of sedation or vasopressor use due to insufficient number of matched pairs, reflecting a limitation of this approach. Infection source, time period of admission, COVID-19 status, and fentanyl exposure were not included in the PSM model and contribute residual confounding effects. Time to blood culture clearance data was only available in about one-third of our patient cohort and, thus, remains a limited surrogate for bacterial dissemination.

## Conclusions

In this retrospective matched cohort, propofol-containing sedation was not associated with fewer ICU-free days, poorer prognosis, or a greater rate of re-infection among mechanically ventilated patients with sepsis or septic shock. Residual confounding and limited precision preclude exclusion of clinically important benefit or harm. Future prospective clinical trials are essential to fully elucidate the immunomodulatory effects of propofol sedation, if any, among mechanically ventilated septic patients.

## Figures and Tables

**Figure 2:**
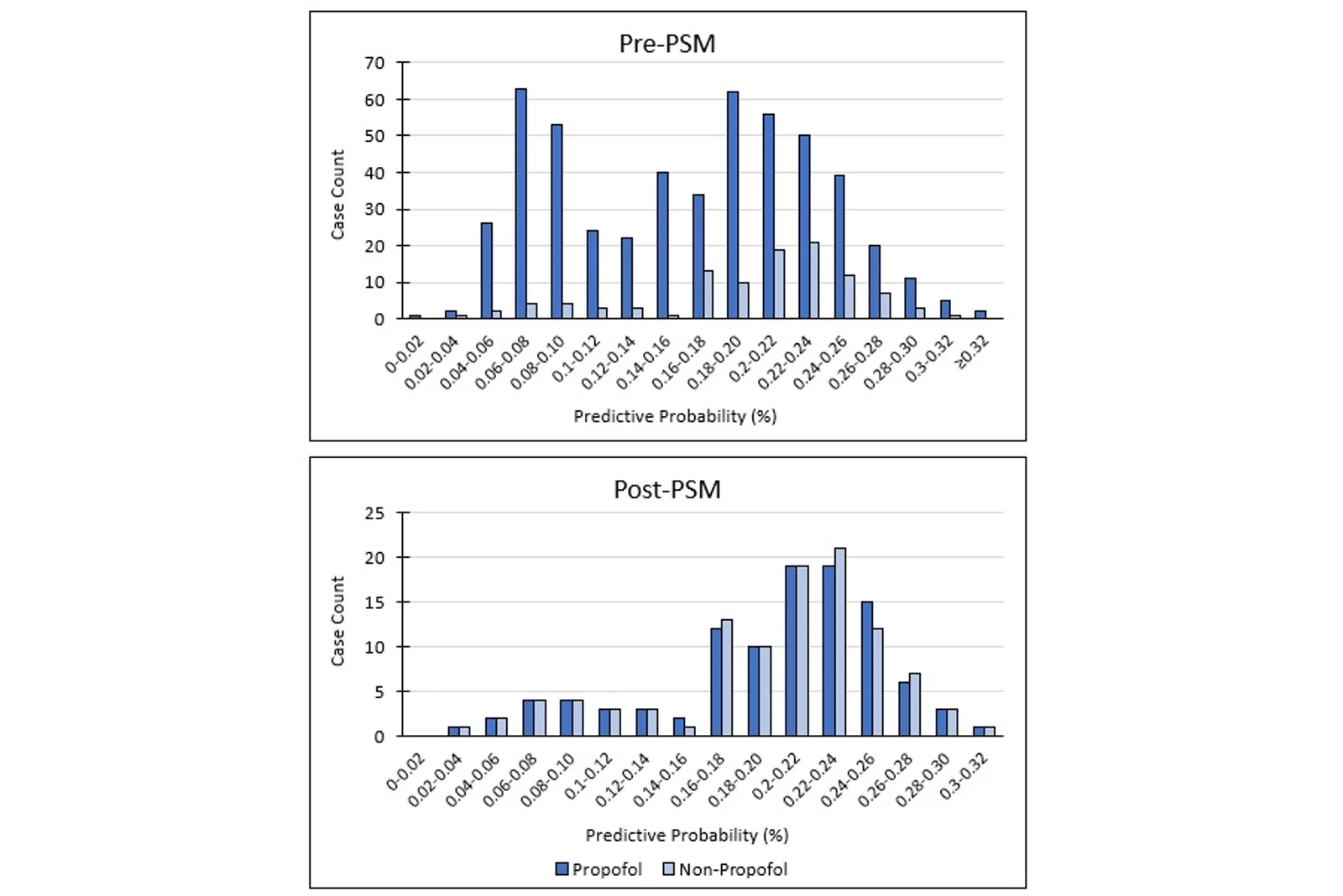
Predictive Probability PSM, Propensity Score Matching

## Appendices

 Appendix A

**Table 8:** ICD-10 Codes for Sepsis and Septic Shock ICD-10, International Classification of Diseases (Tenth Revision)

Sepsis	Septic Shock
A02.1	R65.21
A20.7	T81.12 (XA, XD, XS)
A22.7	
A26.7	
A32.7	
A40 (0.0, 0.1, 0.3, 0.8, 0.9)	
A41 (0.01, 0.02, 0.1, 0.2, 0.3, 0.4, 0.5, 0.51, 0.52, 0.53, 0.59, 0.81, 0.89, 0.9)	
A42.7	
A54.86	
B37.7	
O03 (0.37, 0.87)	
O04.87	
O07.37	
O08.82	
O85	
O86.04	
R65.20	
T81.44 (XA, XD, XS)	

Appendix B
